# Methods of conduct and reporting of living systematic reviews: a protocol for a living methodological survey

**DOI:** 10.12688/f1000research.18005.2

**Published:** 2019-07-16

**Authors:** Assem M. Khamis, Lara A. Kahale, Hector Pardo-Hernandez, Holger J. Schünemann, Elie A. Akl

**Affiliations:** 1 Clinical Research Institute, American University of Beirut, Beirut, Lebanon; 2 AUB GRADE Center, American University of Beirut, Beirut, Lebanon; 3 Julius Center for Health Sciences and Primary Care, Utrecht University, Utrecht, The Netherlands; 4 Iberoamerican Cochrane Centre, Sant Pau Biomedical Research Institute, Barcelona, Spain; 5 CIBER Epidemiología y Salud Pública, Barcelona, Spain; 6 Department of Medicine, McMaster University, Hamilton, Canada; 7 Department of Health Research Methods, Evidence and Impact, McMaster University, Hamilton, Canada; 8 Department of Internal Medicine, American University of Beirut, Beirut, Lebanon

**Keywords:** living systematic review, research methodology, research reporting, study protocol

## Abstract

**Background: **The living systematic review (LSR) is an emerging approach for improved evidence synthesis that uses continual updating to include relevant new evidence as soon as it is published. The objectives of this study are to: 1) assess the methods of conduct and reporting of living systematic reviews using a living study approach; and 2) describe the life cycle of living systematic reviews, i.e., describe the changes over time to their methods and findings.

**Methods: **For objective 1, we will begin by conducting a cross-sectional survey and then update its findings every 6 months by including newly published LSRs. For objective 2, we will conduct a prospective longitudinal follow-up of the cohort of included LSRs. To identify LSRs, we will continually search the following electronic databases: Medline, EMBASE and the Cochrane library. We will also contact groups conducting LSRs to identify eligible studies that we might have missed. We will follow the standard systematic review methodology for study selection and data abstraction. For each LSR update, we will abstract information on the following: 1) general characteristics, 2) systematic review methodology, 3) living approach methodology, 4) results, and 5) editorial and publication processes. We will update the findings of both the surveys and the longitudinal follow-up of included LSRs every 6 months. In addition, we will identify articles addressing LSR methods to be included in an ‘LSR methods repository’.

**Conclusion: **The proposed living methodological survey will allow us to monitor how the methods of conduct, and reporting as well as the findings of LSRs change over time. Ultimately this should help with ensuring the quality and transparency of LSRs.

## Background

The living systematic review (LSR) is an emerging approach for evidence synthesis that uses continual updating to include relevant new evidence as soon as it is published ^1^. LSR aims to make the relevant evidence available to users, soon after its publication. This could lead to “living knowledge translation” in the form of living guideline recommendations ^2^ and living support systems to clinical and policy decisions ^3^. Brazinova *et al.* and Cnossen *et al.* published in 2016 two of the earlier LSRs ^4, 5^. The first series of LSR in Cochrane were published starting in 2017 ^6^. LSRs are expected to take advantage of their currency to enhance the accuracy and utility of evidence synthesis ^3, 7^. Given their appeal, there appears to be an increase in the number of LSRs being conducted and published.

Conducting LSRs requires many of the steps of conducting traditional systematic reviews. However, they are more likely than traditional reviews to benefit from enabling technologies, including but not limited to automatic retrieval of full-text papers or for machine learning-assisted risk of bias assessment ^8^. In addition, LSRs require steps that are specific to the living approach, such as frequent searches or protocols for triggering meta-analyses updating ^9^. On the other hand, LSRs may face some specific challenges, including statistical problems with frequent updating of meta-analyses ^9^, need for sustained funding, and the ability of the publication platform to allow frequent updates.

Reporting LSRs should in principle adhere to all the elements of traditional systematic reviews, as detailed in the Preferred Reporting Items for Systematic Reviews and Meta-Analyses (PRISMA) statement ^10^. However, LSRs’ protocols and final reports should also reflect the methodological features specific to their living approach. For example, LSR reports should highlight the rationale for choosing a living approach over a traditional approach. In addition LSR reports should describe the planned frequency of updating ^1^, the editorial process, and the transition from a traditional to a living systematic review, if applicable ^1^.

While this is still an emerging field, we are not aware of any systematic assessments of the methodological approaches and reporting practices for LSRs. Such assessment would help with better understanding of the conduct and reporting of LSRs and in improving and standardizing them. That would ultimately help with ensuring the quality and transparency of LSR.

## Study objectives

The objectives of this study are to:

1. Assess the methods of conduct and reporting of living systematic reviews;
2. Describe the life cycle of living systematic reviews, i.e., describe the changes over time to the methods and findings of living systematic reviews.

## Methods

### Definitions

We had defined living systematic reviews (LSR) as: “a systematic review that is continually updated, incorporating relevant new evidence as it becomes available.” ^1^.

- We distinguish between the base LSR (which refers to the first published version of the LSR) and the subsequent LSR updates.
- The operational criterion defining the eligibility of a LSR will be as follows: authors label their study as a ‘living systematic review’ (using this or similar terminology).
- We will consider that an LSR lost its living status when its authors report it as such or when they fail to publish an update after a period that is triple that of the planned updates, according to the LSR protocol.

We define a ‘living methodological survey’ (LMS) as a study examining a specific aspect of research methodology (e.g., conducting or reporting of studies), with the findings of this survey being continually updated, incorporating relevant new data as they become available. The aim of a LMS is to reflect the current status of the research methodology aspect being assessed. The study involves no human subjects and requires no ethical approval.

### Overall study design

We will conduct a LMS of LSRs. To address the first objective (i.e., assessing the methods of conduct and reporting of LSRs), we will first conduct a cross-sectional survey of studies that used LSR methodology (the ‘base LMS’). Then, we will update the cross-sectional surveys at regular time intervals (every 6 months) by including LSRs published since the previous update (the ‘LMS updates’). The LMS update will exclude from its analysis a previously included LSR that loses its living status.

To address the second objective (i.e., describing the changes over time to the methods and findings of LSRs), we will conduct a prospective longitudinal follow-up of the cohort of all LSRs identified by the first study. The aim will be to describe the changes over time of the methods and findings of included published LSRs (e.g., frequency of updating, cessation of the living approach, adaptation to newly emerging technologies).

### Eligibility criteria

We will include studies labeled as ‘living systematic reviews’ addressing a health topic, irrespective of the health field (i.e., basic sciences, clinical, public health, health policy and systems), date or language of publication. We will include both ongoing LSRs and LSR protocols. We will also include studies addressing LSR methods and include them in an ‘LSR methods repository’. We will include all LSRs regardless of the design of included studies (e.g., RCTs or observational studies) or of whether they are published in a scientific journal or not.

### Search strategy

We will search the following electronic databases: Medline, EMBASE and the Cochrane library. The search strategy uses both key words and MeSH terms judged to be relevant to our topic (Extended data 1). We developed our search strategy with the help of a librarian experienced (Ms. Aida Farha at the American University of Beirut) in systematic review methodology. We used studies identified by a pilot search to refine the search strategy. We will set the alerts in the databases to search for newly published LSR and will follow for update(s) of the already included LSR. We will also search for LSRs in the Cochrane Library, the Epistemonikos database, as well as journals known (or found through this study) to publish LSRs. Lastly, we will contact groups conducting LSRs to identify eligible that we might have missed including those not published in scientific journals.

### Article selection

Reviewers will complete calibration exercises and then screen in duplicate and independently the titles and abstracts of citations identified by the search. We will obtain the full texts of any citations judged as potentially eligible by at least one reviewer. Reviewers will subsequently screen in duplicate and independently the full texts. They will check agreement and resolve any disagreements by discussion and involve a third review author as needed. We will record reasons for exclusion and summarize the results of the selection process using a PRISMA flow diagram. We will repeat this process for each LMS update.

### Data abstraction

We developed and pilot-tested a standardized data extraction form with detailed instructions (Extended data 2). Reviewers will complete calibration exercises and then extract data in duplicate and independently. They will compare results and resolve disagreements through discussion, or with the help of a third reviewer as needed. We will collect and manage study data using Research Electronic Data Capture (REDCap) tool hosted at the American University of Beirut. REDCap is a secure, web-based application designed to support data capture for research studies. We will export abstracted data from REDCap for every update our analysis.

For each included LSR, we will abstract information for each update on the following:

  1.   General characteristics ( Table 1):

- If the publication is a LSR protocol, base LSR or LSR update (and its number);
- Protocol: if referred to, registered, published; or modified;
- Type of field: basic sciences, clinical, health systems and policy, public health;
- Date of publication (year and month);
- Whether it is a Cochrane review
- Number of authors (total, newly added, newly removed);
- Whether or not authors changed from previous version in terms of ranking/role (first author, last author, and corresponding author);
- Funding (type of funding: continuing vs. expired vs. new funding; source of funding; reporting on the role of funder);
- If and how conflicts of interest were reported.

**Table 1.** General characteristics of the living systematic review (LSR) publication.

Publication type
LSR protocol (%)
Base LSR (%)
LSR update (%)
Protocol (applies to base LSR or LSR update)
Referred to (%)
Registered (%)
Published (%)
Modified (%)
Type of field
Basic sciences (%)
Clinical (%)
Health systems and policy (%)
Public health (%)
Other (%)
Year of publication (range)
Cochrane review (%)
Number of authors
Total (median [IQR])
Newly added (median [IQR])
Newly removed (median [IQR])
Authorship change since previous version
First author (%)
Last author (%)
Corresponding author (%)
Type of Funding
New (%)
Continuing (%)
Expired (%)
Source of funding
Private for profit (%)
Private not-for-profit (%)
Government (%)
Other (%)
Reported on the role of funder (%)
Conflicts of interest reported (%)

IQR – interquartile range

  2.   Systematic review methodology ( Table 2, Table 3 & Table 4):

- If the LSR builds on a previously published traditional SR;
- Type of eligible primary studies (e.g., trials, non-randomized studies);
- Rating certainty
- Summary of findings (SoF) tables
- Tools and/or platform used for SR and output authoring (e.g., RevMan, GRADEpro);
- Use of task sharing processes (e.g., crowd participation);
- Use of machine assisted production processes (e.g., Cochrane RCT classifier);
- The SR includes network meta-analysis;
- Quality of reporting, using the PRISMA checklist ^10^;
- Quality of conduct, using the AMSTAR 2 tool ^11^.

**Table 2.** Systematic review methodology.

LSR builds on a previously published traditional SR (%)
Type of eligible primary studies
Randomized clinical trials (%)
Nonrandomized trials (%)
Observational studies (cohort, Case-control, cross-section) (%)
Case studies and case series (%)
Rating certainty
SoF tables
Tools and/or platform used for SR authoring
Review Manager (%)
Other (%)
Use of task sharing processes (%)
Cochrane crowd (%)
Other (%)
Use of machine assisted SR production processes
Cochrane RCT classifier (%)
Other (%)
SR includes network meta-analysis (%)

LSR – living systematic review, SR – systematic review, SoF – summary of findings, RCT – randomized clinical trial

**Table 3.** Quality of reporting using PRISMA checklist.

	1	2	3	4	5	6	7	8	9	10	11	12	13	14	15	16	17	18	19	20	21	22	23	24	25	26	27
**LSR1**																											
**LSR2**																											
**LSR3**																											
**LSR4**																											

LSR – living systematic review

**Table 4.** Quality of conduct using AMSTAR 2 tool.

	1	2	3	4	5	6	7	8	9	10	11	12	13	14	15	16
**LSR1**																
**LSR2**																
**LSR3**																
**LSR4**																

LSR – living systematic review

  3.   Living approach methodology ( Table 5):

- Rationale for LSR provided (priority, uncertainty, emerging evidence, other);
- Method of literature surveillance; sources (newly added, newly removed, retained); use of auto alerts; use of traditional search updates; search frequency, modification of search terms;
- Use of meta-analytic methods to adjust for frequent updating (e.g. trial sequential analysis, sequential meta-analysis, the Shuster method, Law of the iterated logarithm);
- Changes in LSR methodology compared to the previous version of the LSR.

**Table 5.** Living approach methodology.

Rationale for LSR provided:
Priority (%)
Uncertainty (%)
Emerging evidence (%)
Other (%)
Number of sources
Newly added (median [IQR])
Retained (median [IQR])
Newly removed (median [IQR])
Use of auto alerts (%)
Use of traditional search update (%)
Search frequency (median [IQR]) in months
Search terms modified (%)
Meta-analytic methods adjusted for frequent updating
Trial sequential analysis (%)
Sequential meta-analysis (%)
The Shuster method (%)
Law of the iterated logarithm (%)
Not adjusted (%)
Changes in LSR methodology compared to the previous version of the LSR (%)

LSR – living systematic review, IQR – interquartile range

  4.   LSR results ( Table 6):

- Elements of the PICO question modified;
- Number of the LSR version;
- Time since preceding update;
- Number of citations screened for the LSR update period;
- Number of identified newly published eligible primary study protocols;
- Number of identified newly published eligible primary studies;
- Dealing with identified newly published eligible primary studies (i.e., incorporated or not);
- Change in statistical results, change in certainty of evidence, and change in conclusions.

**Table 6.** Results of living systematic reviews (LSRs).

Elements of the PICO question modified
Number of the LSR version (median [IQR])
Time since publication of previous version (median [IQR]) in months
Number of citations screened the LSR update period (median (IQR))
Number of identified newly published eligible primary study protocols (median (IQR))
Number of identified newly published eligible primary studies (median (IQR))
Dealing with identified newly published eligible primary studies
No new evidence (%)
New evidence, but not incorporated (%)
New evidence incorporated (%)
Change in statistical results (%)
Change in certainty of evidence (%)
Change in conclusions (%)

PICO – patient intervention comparison outcome, IQR – interquartile range

  5.   Editorial and publication processes ( Table 7):

- Whether the LSR version has been peer reviewed or not;
- Whether the managing editor and the peer reviewers are the same as for the previous version;
- Time required for the editorial and peer review processes;
- Journal (or platform) of publication; its impact factor; whether open access; whether it provides instructions for reporting of systematic reviews, and for LSRs respectively;
- Whether the journal (or platform) accommodates iterative versions of the same document (e.g., nano-publication approach, sub-doi);
- Approach to flagging changes in methods and findings for reader (new evidence).

**Table 7.** Editorial and publication processes.

LSR version peer-reviewed (%)
The managing editor is the same as for the previous version (%)
Peer reviewers are the same as for the previous version (%)
Time required for editorial and peer review processes (median (IQR)) in months
Published in open access (%)
Journal impact factor (median (IQR))
Journal provides instructions for reporting of systematic reviews (%)
Journal provides instructions for reporting of LSRs (%)
Journal/platform accommodate iterative versions of the same document
Use of nano-publication approach (%)
Sub-doi (%)
Other (%)
Approach to flagging new evidence for reader (%)

LSR – living systematic review, IQR – interquartile range

### Data analysis plan

**Figure 1.**
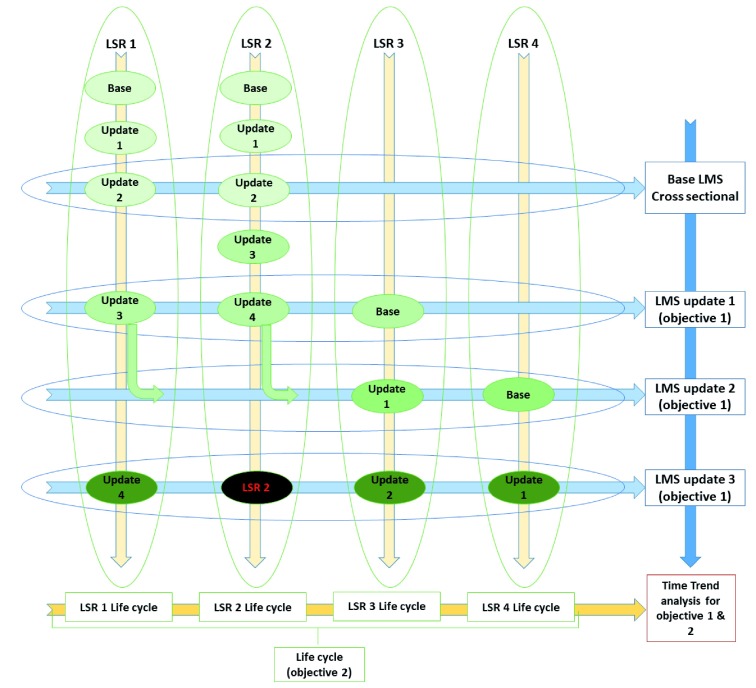
the data analysis plan for the living methodological survey (LMS) and longitudinal follow-up of living systematic reviews (LSRs).

We will perform separate data analyses for two objectives included in this LMS ( Figure 1):

1. Cross sectional survey: For each LMS update, we will run a summary descriptive analysis of the variables of interest (related to conduct and reporting of LSRs) across the latest versions of included LSRs. With each LMS update, we will update the summary descriptive analysis, and archive the results of the previous LMS update. We will exclude from the cross sectional surveys the reviews that have lost their living mode status. We will run an analysis and present a graphical presentation of selected variables over time to show trends of changes in all LSRs in our survey updates, which adds a longitudinal dimension to this objective.
2. Longitudinal follow-up: For each LSR, we will analyze the changes over time (i.e., the LSR lifetime) of selected variables (related to methods and results). We graphically present the findings to show their time trends.

## Dissemination

We will publish the study protocol and the living methodological survey (LMS) in *F1000Research* journal. *F1000Research* journal has a dynamic publication process that allows adding versions of the LMS that represent the six-monthly updates, while making copies of the previous updates available.

## Study status

We have finalized the search strategies for Medline, EMBASE, and the Cochrane library. We have drafted the data abstraction form. We are planning to launch the study after the protocol is published.

## Discussion

The main objectives of this LMS are to assess the methods of conduct and reporting of LSRs and describe the changes over time to their methods and findings (the life cycle of living systematic reviews). This is the first methodological study that follows a living approach and that continuously surveys the methods of conduct, and reporting of LSRs. We aim to add newly published LSRs soon after their publication. This will ensure that our findings will be both current and representative of published LSRs.

We foresee that our LMS may be limited by the fact that we will focus on ongoing or published LSRs. As such, we may miss newly developed LSR methodologies that are being tested but not reported as of yet. We hope to overcome this shortcoming by surveying Cochrane groups and authors of LSRs on their unpublished LSR initiatives. Similarly, we might not obtain needed information from previous versions of published LSRs (i.e., when we run our first search) and therefore be unable to capture previous methodological approaches. Lastly, we might face some challenges in analyzing and presenting the time trends of our findings, since we expect heterogeneity in the field given the novelty of the approach. Maintaining our LMS in the living mode will require sustained efforts and resources.

The proposed LMS will allow us to monitor how the methods of conduct, and reporting as well as the findings of LSRs will change over time. In addition, we will be able to pinpoint potential gaps and research needs in the field of LSRs. We hope this survey will advance the methodology and subsequently the quality of LSRs, fostering in turn the currency of evidence supporting decision making for practice and policies. The findings of this survey may inform the development of an extension for PRISMA statement for LSR. Furthermore, it might help in the advancement of editorial and publication processes of LSR.

## Data availability

### Underlying data

No data is associated with this article.

### Extended data

Figshare: Extended data 1. Search strategies, https://doi.org/10.6084/m9.figshare.7688036 ^12^

Figshare: Extended data 2. LSR_data abstraction form_20190129.xlsx, https://doi.org/10.6084/m9.figshare.7687823 ^13^

Data are available under a Creative Commons Attribution 4.0 International (CC BY 4.0) license
