# Supplementary material for: Methods of conduct and reporting of living systematic reviews: a protocol for a living methodological survey
Source: F1000Res. 2019 Jul 16;8:221. Originally published 2019 Feb 26. [Version 2] doi: 10.12688/f1000research.18005.2 (PMC6556985; doi:10.12688/f1000research.18005.2)
Supplement: Peer review history

# Peer review: Reviewer response for version 1

**DOI:** 10.5256/f1000research.19692.r48191

**Published:** 2019-06-07

**Authors:** Terri Pigott

**Affiliations:** 1 Loyola University Chicago, Chicago, IL, USA

This protocol describes methods for the conduct of a living systematic review focused on the methods for living systematic reviews. The objectives of the study will be to assess the methods of conduct and reporting of living systematic reviews and to describe changes over time to living systematic review methods and findings. The research will include a cross-sectional survey of the methods and findings of living systematic reviews that will be updated every six months to include newly published living systematic reviews. The research will also analyze the changes over time in the methods and results used in the included living systematic reviews.

 The study will make an important contribution. When new methods such as those for living systematic reviews are introduced in the literature, researchers wanting to use the method may have difficulty accessing articles and guidance. A living systematic review that documents methods for these types of systematic reviews gives researchers access to the most current applications of this method. These methods are also developing rapidly in the literature and living systematic reviews serve to keep the field aware of how researchers are applying the methods. As described in the protocol, living systematic reviews are more likely to use emerging technologies in the field such as machine learning-assisted methods. A living systematic review demonstrating the use of these emerging technologies is another potential contribution of the research.

 The study methods are appropriate and use best practice standards for systematic review. The protocol does not provide a sense of the number of these reviews that are currently being conducted so it is not clear how large a review will result from this research. Sufficient details are provided to allow replication by other researchers.

I confirm that I have read this submission and believe that I have an appropriate level of expertise to confirm that it is of an acceptable scientific standard.

# Peer review: Reviewer response for version 1

**DOI:** 10.5256/f1000research.19692.r48749

**Published:** 2019-05-22

**Authors:** Laurence Le Cleach

**Affiliations:** 1 Department of Dermatology, Universite Paris-Est, Creteil, France; 2 d’Accueil EA 7379, EpiDermE (Epidemiology in Dermatologie and Therapeutics evaluation), Université Paris-Est, Creteil, France

The topic of this protocol is interesting and important. LSR is an emerging method and this study will contribute to enhance its methodology and publication process.

**Major remarks**

 The background could be developed in order to describe more precisely the issues, methodological difficulties, funding and publication of the LSR. When the first one was published? How many are currently published, are they published in scientific journals? For these last questions it is one of the aims of the study but authors should have an idea at least in the Cochrane library.

 In the discussion, other goals could be described such as contributing to an extension of PRISMA for LSR, providing information to contribute to build another editorial and publication process for living SR? Build collaboration with teams identified to collectively share tools, skills or enhanced methodology?

**Minor remarks**

- The author stated "a librarian experienced in systematic review methodology". Could they provide his/her name and affiliation.
- The authors stated in Abstract "We will also contact groups conducting LSRs to identify eligible studies that we might have missed" and in Methods: "Lastly, we will contact groups conducting LSRs to identify eligible that we might have missed". Indeed, In SR methodology it is normal to contact experts in the field and authors to obtain additional studies potentially missed. But in this setting is it relevant?
- The authors stated "Then, we will update the cross-sectional surveys at regular time intervals (e.g., 6 months) by including LSRs published since the previous update (the ‘LMS updates’)". Does this mean that you have not decided yet the frequency of your updates?
- Could you discuss that considering the methods and tools used if you are focused on RCT Living SR?
- Will this protocol be registered in Prospero as well?
- Could you explain why you chose to use AMSTAR rather than ROBIS?
- Could you explain how do you plan to published updates each 6 months?

I confirm that I have read this submission and believe that I have an appropriate level of expertise to confirm that it is of an acceptable scientific standard.
